# Analysis of gastroscopy results among healthy people undergoing a medical checkup: a retrospective study

**DOI:** 10.1186/s12876-020-01557-9

**Published:** 2020-12-09

**Authors:** Haosu Huang, Yanting Rong, Meng Wang, Zimeng Guo, Yanghua Yu, Zhenpu Long, Xiaoxiao Chen, Hanyue Wang, Junjie Ding, Lu Yan, Jie Peng

**Affiliations:** Department of Gastroenterology, Xiangya Hospital, Central South University, 87 Xiangya Road, Changsha, 410008 Hunan Province China

**Keywords:** Abnormal upper gastrointestinal endoscopic results, Gastroscopy, Health check, Screening

## Abstract

**Background:**

The association of upper gastrointestinal endoscopic findings with sex, age, and *Helicobacter pylori* infection in asymptomatic healthy people is unclear. The aim of this study was to retrospectively determine the associations of upper gastrointestinal endoscopic findings in asymptomatic healthy people with sex, age, and *H. pylori* infection.

**Methods:**

A retrospective study was conducted on 2923 patients from a health examination center in Xiangya Hospital between September 2015 and September 2019. Data on sex, age, *H. pylori* infection, and gastroscopy results were collected.

**Results:**

Among 2923 asymptomatic patients who underwent gastroscopy, 2911 (99.59%) had abnormal results. The top three results were chronic gastritis (95.11%), peptic ulcer (17.45%), and duodenitis (9.17%). Inflammation of the gastric mucosa in chronic gastritis was more severe in the *H. pylori*-positive group. The incidence of peptic ulcer decreased with increasing age and was higher in men, patients aged < 30 years, and *H. pylori*-positive patients. The incidence of polyps was higher in women (9.54%) than in men (5.94%), and the incidence in individuals aged ≥60 years (11.63%) was higher than that in those aged < 60 years (6.83%). The pathological results of gastric polyps depended on the location of the lesion.

**Conclusion:**

The incidence of abnormal upper gastrointestinal endoscopic results is high in asymptomatic healthy people undergoing a check-up and is associated with sex, age, and *H. pylori* infection. Gastroscopy should be considered part of a routine health check.

## Background

Physical examination in the outpatient setting is a valuable tool. Even in settings where there is a lack of evidence, such as the annual physical examination of the asymptomatic adult, physical examination is beneficial for the physician-patient relationship. When a patient has specific symptoms, physical examination can help narrow down or, in many cases, establish a diagnosis [1]. Physical examination is an important feature of healthcare encounters and is considered a key aspect of diagnosis and treatment planning [2]. In an annual physical examination, imaging and laboratory tests are easily available, including gastroscopy.

Gastroscopy enables physicians to visualize a variety of upper gastrointestinal lesions, particularly small lesions [3]. Employing gastroscopy is essential for the diagnosis of gastric and upper gastrointestinal diseases. Nevertheless, gastroscopy is generally indicated only in case of symptomatic patients. In asymptomatic healthy people, routine endoscopy remains controversial. As the pace of modern life continues to accelerate, the pressures from work, family, and society are constantly increasing. People find it difficult to maintain good eating patterns, and the phenomenon of overeating is common. With the ever growing economic development of society, gastroscopy is becoming increasingly common as a physical examination item; screening for upper gastrointestinal lesions have therefore become a regular part of medical examinations. In the context of routine physical examinations, it is possible to screen for abnormal upper gastrointestinal endoscopic results and to achieve early detection, prevention, and diagnosis.

In this study, we sought to clarify the incidence of various abnormal upper gastrointestinal endoscopic results and to compare how they varied according to sex and age in healthy, asymptomatic people undergoing a check-up. Moreover, we also evaluated the association between the incidence of abnormal endoscopic results and *Helicobacter pylori* (Hp) infection, as well as the association between pathological results and lesion location.

## Methods

### Subjects

This retrospective study included the results from healthy individuals age 14–78 years who underwent gastroscopy at a health examination center at Xiangya Hospital (a tertiary endoscopic center) between September 2015 and September 2019. There were 2923 subjects (1718 men and 1205 women). The mean age was 46.4 ± 10.3 years. Subjects were divided into five subgroups according to age (< 30 years, 30–39 years, 40–49 years, 50–59 years, and ≥ 60 years). The study protocol was approved by the Ethic Committee of the Xiangya Hospital of Central South University (No: 201912541). Instruments used included an Olympus-260 endoscope. Patients were fasted for 8 h and water deprivation was performed for 4 h before treatment. Oral administration of dacronine hydrochloride (10 ml:0.1 g) was performed before gastroscopy for mucosal lubrication and anesthesia. Patients who underwent painless gastroscopy were given balanced anesthesia before gastroscopy. Balanced anesthesia was not performed for patients who underwent ordinary gastroscopy.

### Inclusion criteria

The subjects who were referred for gastroscopy over a 4-year period (September 2015 to September 2019) and had complete basic information were eligible for the study. The health examination center sent questionnaires to patients before the health check, including filling in uncomfortable symptoms and medical history. Asymptomatic individuals were determined according to the questionnaire’s results and systemic physical examination. At the outset, we excluded patients with symptoms of the upper digestive tract, including acid reflux, belching, or heartburn and those who had a definite diagnosis of upper gastrointestinal disease.

### Data collection

The basic data collected at the endoscopy center included sex, age, detection of Hp, and gastroscopy results. The pathological examination provided data regarding gastroscopy diagnosis, biopsy site, and pathological diagnosis.

### Diagnosis

Current practice guidelines advocate testing patients for Hp infection primarily using non-invasive methods, including the urea breath test (UBT), and invasive methods, including histology examination [4]. Hp tests in the study included UBT and histological examination of specimens obtained using gastroscopy. For clinical diagnosis, 2–3 pieces of tissue were taken for biopsy at the gastric antrum, lesser curvature of stomach, and gastric body sites. Biopsy was also taken at the suspected lesion. Endoscopy combined with histopathological examination can diagnose the two basic types of chronic gastritis: chronic non-atrophic gastritis and chronic atrophic gastritis. The endoscopic findings of chronic non-atrophic gastritis included mucosal erythema, bleeding mucosal spots or plaques, and mucosal roughness, either with or without edema, congestion, or exudation. The endoscopic findings of chronic atrophic gastritis included mixed red and white shades of mucosa dominated by white mucosa, flat or even absent folding, and exposure of part of the mucosal blood vessels [5]. The final diagnosis of chronic atrophic gastritis was based on the pathological diagnosis. The pathologic diagnosis of chronic gastritis was in accordance with the Chinese consensus on chronic gastritis (2017, Shanghai) [6]. The criteria of histopathological grading are as follows: in mild cases, the lamina propria of the mucosa is infiltrated with few neutrophils; in moderate cases, more neutrophils are seen in the mucosal layer and can also be seen in between superficial epithelial cells, pit epithelial cells and glandular epithelial cells. In severe cases, denser infiltration of neutrophils, or abscess on pits can be seen in addition to what is seen in moderate activity. Other upper gastrointestinal lesions were diagnosed according to relevant guidelines.

### Statistical analysis

SPSS 26.0 statistical software (SPSS, Inc., Chicago, IL, USA) was used to perform the statistical analyses. Measurement data are expressed as mean ± SD, and comparisons between groups were performed using t-tests. Countable data were analyzed using the χ^2^ test. *p* < 0.05 indicated statistically significant differences.

## Results

### Overall results for abnormal upper gastrointestinal endoscopic results

Normal upper gastrointestinal endoscopic results were observed in 12 patients (0.41%; 4 men and 8 women). The remaining 2911 patients (99.59%) had abnormal upper gastrointestinal endoscopic results (1714 men and 1197 women). There were 184 patients in the < 30 years age group, 462 patients in the 30–39 years age group, and 1100 patients in the 40–49 year age group. There were 816 patients aged 50–59 years, and 361 patients older than 60 years. A total of 976 Hp tests were completed and 354 cases were positive (36.27%).

### Distribution of lesions detected in the upper digestive tract

Among 2923 patients who underwent gastroscopy, the five most common abnormal endoscopic results were as follows (Table 1): Chronic gastritis (95.11%), peptic ulcers (17.45%), duodenitis (9.17%), esophagitis (8.96%), and polyps (7.42%) . Raised lesions were examined in 70 cases (2.39%). Diseases such as gastric retention (0.44%), esophageal varices (0.24%), and ectopic pancreas (0.17%) were rare (Table 1).

**Table 1:** Distribution of lesions detected in the upper gastrointestinal tract

Abnormal upper gastrointestinal endoscopic results	Number of cases detected (%)
Chronic gastritis	2780 (95.11)
Peptic ulcers	510 (17.45)
Duodenitis	268 (9.17)
Esophagitis	262 (8.96)
Polyps	217 (7.42)
Raised lesions to be examined	70 (2.39)
Esophageal papilloma	36 (1.23)
Gastric retention	13 (0.44)
Esophageal leiomyoma	11 (0.38)
Upper gastrointestinal diverticulum	10 (0.34)
Gastric cancer	9 (0.31)
Esophageal varices	7 (0.24)
Ectopic pancreas	5 (0.17)
Achalasia	4 (0.14)
Esophageal cancer	3 (0.10)
Gastric lymphoma	2 (0.07)
Heterosis of esophagus and stomach mucosa	1 (0.03)
Esophageal glycogenic acanthosis	1 (0.03)

## Analysis of factors related to abnormal upper gastrointestinal endoscopic results

### Relationship between abnormal upper gastrointestinal endoscopic results and sex

#### Relationship between chronic gastritis and sex

Among 2780 cases of chronic gastritis, there were 1635 men and 1145 women (95.17 and 95.02%, respectively) (Table 2). The occurrence of chronic gastritis was higher in men than in women, although the difference was not significant (χ^2^ = 0.03, *p* = 0.862).

**Table 2:** Relationship between abnormal upper gastrointestinal endoscopic results and sex

Sex	Gastroscopy results		Total	χ^2^	*P*-value
	Chronic gastritis	Non-chronic gastritis			
Male	1635	83	1718		
Female	1145	60	1205	0.03	0.862
Total	2780	143	2923		

Sex	Gastroscopy results		Total	χ^2^	*P*-value
	Peptic ulcer	Non-peptic ulcer			
Male	376	1342	1718		
Female	134	1071	1205	56.988	0.000
Total	510	2413	2923		

Sex	Gastroscopy results		Total	χ^2^	*P* value
	Polyps	Non-polyps			
Male	102	1616	1718		
Female	115	1090	1205	13.403	0.000
Total	217	2706	2923		

#### Relationship between peptic ulcer and sex

Among 510 cases of peptic ulcers, seven were esophageal ulcers and the remainder were gastric and duodenal ulcers. Peptic ulcers occurred in 376 men and 134 women (21.89 and 11.12%, respectively) (Table 2). The occurrence of peptic ulcer was higher in men than in women (χ^2^ = 56.988, *p* = 0.000).

#### Relationship between polyps and sex

Among 217 patients with polyps, there were 102 men and 115 women (5.94 and 9.54%, respectively) (Table 2). The occurrence of polyps was higher in women than in men (χ^2^ = 13.403, *p* = 0.000).

### Relationship between abnormal upper gastrointestinal endoscopic results and age

#### Relationship between peptic ulcer and age

A total of 510 patients with peptic ulcers were identified (Table 3); the occurrence of peptic ulcer gradually decreased with increasing age. We made independent comparisons between each age group (Table 3), and we adjusted αˊ to 0.005(αˊ = 2α/(k(k-1)) according to the Bonferroni correction [7]. There were no significant differences among the age groups (*p* > 0.005). The age groups were further divided into an age group of < 30 years and one of ≥30 years (Table 3). The detection rates of these two groups were 24.46 and 16.98% respectively, with a statistically significant difference between these two groups (χ^2^ = 6.697, *p* = 0.012). Peptic ulcers occurred more frequently in the age group < 30 years than in people ≥30 years.

**Table 3:** Relationship between abnormal upper gastrointestinal endoscopic results and age

Age	Gastroscopy results		χ^2^	*P* value
	Peptic ulcer	Non-peptic ulcer		
30-39 years	89	373	1.053	0.310
40-49 years	188	912		
30-39 years	89	373	1.510	0.221
50-59 years	135	681		
30-39 years	89	373	2.981	0.094
60 years and above	53	308		
40-49 years	188	912	0.100	0.758
50-59 years	135	681		
40-49 years	188	912	1.146	0.290
60 years and above	53	308		
50-59 years	135	681	0.647	0.639
60 years and above	53	308		
Under 30 years	45	139	6.697	0.012
30 years and above	465	2274		

Age	Gastroscopy results		χ^2^	*P* value
	Polyps	Non-polyps		
Under 30 years	19	165	2.406	0.144
30 years and above	198	2541		
Under 40 years	57	589	2.364	0.126
40 years and above	160	2117		
Under 50 years	137	1609	1.127	0.314
50 years and above	80	1097		
Under 60 years	175	2387	10.624	0.002
60 years and above	42	319		

#### Relationship between polyps and age

There were 175 patients with polyps in the < 60 years age group (6.83%) and 42 in the ≥60 years age group (11.63%) (χ^2^ = 10.624, *p* = 0.002; Table 3). The detection rate of polyps was higher in the ≥60 years age group than in the < 60 years age group.

### Relationship between abnormal upper gastrointestinal endoscopic results and Hp infection

#### Relationship between chronic gastritis and Hp infection

Among the 2923 patients who underwent medical examinations, a total of 976 were tested for Hp infection. There were 306 cases of chronic gastritis and 48 cases of non-chronic gastritis in the Hp-positive group, with detection rates of 34.19 and 59.26%, respectively (Table 4). Positive Hp infection was only present in 34% of patients with chronic gastritis (statistically significant, χ^2^ = 17.545, *p* = 0.000).

**Table 4:** Relationship between abnormal upper gastrointestinal endoscopic results and *Helicobacter pylori* infection

*H. pylori* test results	Gastroscopy results		Total	χ^2^	*P*-value
	Chronic gastritis	Non-chronic gastritis			
Positive	306	48	354		
Negative	589	33	622	17.545	0.000
Total	895	81	976		
*H. pylori* test results	The extent of gastric mucosal inflammation		Total	χ^2^	*P*-value
	Mild	Moderate or severe			
Positive	26	165	191		
Negative	135	20	155	185.708	0.000
Total	161	185	346		
*H. pylori* test results	Gastroscopy results		Total	χ^2^	*P*-value
	Peptic ulcer	Non-peptic ulcer			
Positive	176	178	354		
Negative	117	505	622	102.583	0.000
Total	293	683	976		
*H. pylori* test results	Gastroscopy results		Total	χ^2^	*P*-value
	Polyps	Non-polyps			
Positive	28	326	354		
Negative	124	498	622	24.815	0.000
Total	152	824	976		
*H. pylori* test results	Pathological types of polyps		Total	χ^2^	*P*-value
	Fundic glandular polyps	Inflammatory polyps			
Positive	7	13	20		
Negative	76	41	117	9.326	0.002
Total	83	54	137		

#### Relationship between the extent of gastric mucosal inflammation and Hp infection in chronic gastritis

The results of 346 gastric mucosal biopsies as follows: there were 26 cases of mild inflammation and 165 cases of moderate or severe inflammation in the Hp-positive group, with detection rates of 13.61 and 86.39%, respectively. There were 135 cases of mild inflammation and 20 cases of moderate or severe inflammation in the Hp-negative group (87.10 and 12.90%) (Table 4). There were significant differences between these two groups (χ^2^ = 185.708, *p* = 0.000). The inflammation of gastric mucosa in chronic gastritis was more severe in the Hp-positive group than in the Hp-negative group.

#### Relationship between peptic ulcer and Hp infection

A total of 976 patients underwent Hp examination. There were 176 Hp-positive and 117 Hp-negative cases with peptic ulcers, with detection rates of 49.72 and 18.81%, respectively (Table 4). More patients infected with *Hp* also had peptic ulcers (statistically significant, χ^2^ = 102.583, *p* = 0.000).

#### Relationship between polyps and Hp infection

A total of 152 patients with polyps underwent Hp examination and 28 patients were Hp positive (18.42%). A total of 824 patients without polyps underwent Hp examination and 326 patients were Hp positive (39.56%) (Table 4). The prevalence of *Hp* infection was higher in the patients in whom no polyps were detected (statistically significant, χ^2^ = 24.815, *p* = 0.000).

#### Relationship between pathological types of polyps and Hp infection

Pathological and Hp tests were performed in 147 patients with polyps. Because hyperplastic polyps and adenomatous polyps were detected only rarely, we compared the association between Hp infection and fundic glandular versus inflammatory polyps, with Hp-positive rates of 8.43 and 24.07%, respectively (Table 4). Patients who tested positive for *Hp* were more likely to develop inflammatory polyps than fundic glandular polyps (statistically significant, χ^2^ = 9.326, *p* = 0.002).

#### Relationship between pathological types of polyps and age

Fundic glandular polyps were the most common pathological types of polyps present at all ages, followed by inflammatory polyps; adenomatous polyps were rare (Table 5).

**Table 5:** Relationship between pathological types of polyps and age

Age	Fundic glandular polyps	Inflammatory polyps	Hyperplastic polyps	Adenomatous polyps	Total
Under 30 years	11	6	1	0	18
30-39 years	15	13	2	0	30
40-49 years	30	23	1	0	54
50-59 years	15	5	3	1	24
60 years and above	12	7	1	1	21
Total	83	54	8	2	147

#### Relationship between pathological types of polyps and distribution of lesions

We compared the pathological locations between the gastric fundus, gastric body, gastric antrum, and duodenum, and there were significant differences among these groups (χ^2^ = 44.188, *p* = 0.000) according to Fisher precision testing (Table 6). The pathological types of polyps were associated with the distribution of polyp lesions. Fundic glandular polyps were most prevalent in the gastric fundus and gastric body, while inflammatory polyps were most prevalent in the gastric antrum and duodenum.

**Table 6:** The correlation between pathological types of polyps and the distribution of lesions

	Fundic glandular polyps	Inflammatory polyps	Hyperplastic polyps	Adenomatous polyps	Total	*P*-value
Gastric fundus	37	18	2	1	58	
Gastric body	42	9	4	0	55	
Gastric antrum	2	13	1	0	16	0.000
Duodenum	2	14	1	1	18	
Total	83	54	8	2	147	

## Discussion

Our study provided valuable data based on a large population of healthy individuals (without digestive symptoms) undergoing a check-up, revealing solid data regarding the incidence of abnormal upper gastrointestinal endoscopic results. In addition, we indicated an association between abnormal upper gastrointestinal endoscopic results in healthy people, and sex, age, and Hp infection in Eastern countries. These asymptomatic people, who were found to have abnormal upper gastrointestinal endoscopic results, could undergo preventative treatment in advance; thus, avoiding the development of upper gastrointestinal diseases. We found that the incidence of abnormal upper gastrointestinal endoscopic results was high in healthy asymptomatic people. This incidence was associated with sex, age, and *Hp* infection. In addition, the pathological results of polyps depended on the location of the lesion. These data suggest that gastroscopy should form part of a routine health check.

Gastroscopy plays a major clinical role in the diagnosis of gastric diseases. The detection and diagnosis of upper gastrointestinal diseases through gastroscopic intervention was the most routine procedure [8, 9]. The development of endoscopic technology has led to an increase in the diagnostic rate of early gastric cancer. Several studies indicated a relationship between upper gastrointestinal diseases and associated factors in symptomatic patients, while the associations of abnormal upper gastrointestinal endoscopic results in asymptomatic healthy people with sex, age, and Hp infection remained unclear.

We found that chronic gastritis was the most common abnormal upper gastrointestinal endoscopic result detected in healthy asymptomatic people undergoing a medical checkup, followed by peptic ulcer, duodenitis, esophagitis, and polyps. Chronic gastritis, a chronic inflammatory condition of the gastric mucosa, was one of the most common findings of endoscopy in the general population of Eastern countries [10]. According to the classification and grading criteria of chronic gastritis proposed by the Chinese Society of Digestive Endoscopy [11], chronic gastritis was classified as superficial gastritis, erosive gastritis, and atrophic gastritis according to endoscopic appearance. Each type of gastritis was further classified as mild, moderate, or severe depending on the extent of severity. In the present study, we found that the positivity rate of Hp infection in the group of chronic gastritis was lower than that of patients without chronic gastritis. This may be because of the impact of environmental factors such as a high-salt diet and smoking, as well as the effect of host genetic factors that could cause chronic gastritis in addition to Hp infection [12]. Another study demonstrated that the reason some individuals developed more severe gastritis and progress to disease was multifactorial and included infection by more virulent strains of Hp [13], this could explain our finding that inflammation of the gastric mucosa in chronic gastritis was more severe in the Hp-positive group than in the Hp-negative group.

Peptic ulcer disease is a source of significant morbidity and mortality worldwide. Approximately two-thirds of patients with peptic ulcer disease were asymptomatic [14]. A higher peptic ulcer disease incidence is associated with male sex, smoking, and chronic medical conditions [15, 16]. In addition, Hp and non-steroidal anti-inflammatory drug use were the causes of the vast majority of peptic ulcers [17]. Peptic ulcer disease has also been found to be associated with increasing age [18]. This is consistent with the results of our study in that more patients infected with *Hp* also had peptic ulcers and the occurrence of peptic ulcer was higher in men than in women. Nevertheless, we found that the detection rate of peptic ulcer decreased with age, and peptic ulcers occurred more frequently in the age group < 30 years than in those aged ≥30 years. This suggests that the association between peptic ulcers and age is different in asymptomatic healthy people and patients. The detection rate of peptic ulcers decreased with age and people in the age group of < 30 years should be advised to pay more attention to the possibility of peptic ulcers than people in the other age groups.

Upper gastrointestinal polyps are mainly composed of gastric polyps (GPs), which are considered to be precancerous stages of gastric cancer. The clinical manifestations of GPs were nonspecific, with the majority of polyps occasionally found during gastroscopy examination [19]. It has been reported that the incidence of GPs was higher in women than in men, with a ratio of 1:1.8 to 2.5 [20, 21]. There was a difference in the incidence of GPs between the sexes. It has been reported that duodenal fluid reflux, which may cause gastric mucosal hyperplasia and polyps, is more common in women [22]. However, further studies are needed to determine whether genetic factors, hormones, and other factors might cause differences in GPs between the sexes. These results corroborate those in our study in that the detection rate of polyps was higher in women than in men, with a ratio of 1:1.6.

It has been reported that the detection rate of GPs increased with increasing age [23]. All types of GPs showed a clear-cut age-dependent rise [24]. Our data also suggest that the detection rate of polyps in the age group of ≥60 years was higher than that in the age group of < 60 years. The results of our study suggest that older patients are more likely to suffer from polyps. These findings suggest that the elderly population should be targeted for screening for upper gastrointestinal polyps.

Zheng et al. [22] reported a retrospective study of 2125 patients diagnosed with GP between January 2004 and December 2013, and found that GPs in the gastric antrum and gastric body were the most prevalent in both sexes. Similarly, inflammatory polyps and hyperplastic polyps were the most prevalent in both sexes. However, our results were different because the prevalence of common types of GPs is not the same across different regions of the world. Fundic glandular polyps were the most common type in western countries [25], while hyperplastic polyps were more common in Turkey [26]. In the present study, the most common type of GPs was the fundic glandular polyps followed by inflammatory polyps. Our study also suggests that fundic glandular polyps account for a significant proportion of those found in the gastric fundus and gastric body, and inflammatory polyps account for a significant proportion of those found in the gastric antrum and duodenum. Understanding the occurrence of the various types of GPs is important [27, 28] because gastric adenomatous polyps have a high malignancy potential, and a small number of hyperplastic polyps are cancerous.

A previous study reported that Hp infection was significantly less frequent in all subjects with GPs than in controls [23]. This is consistent with our finding that the positivity rate of Hp in the polyp group was lower than that in the non-polyp group. In another study, it was found that Hp infection rates in patients with inflammatory and hyperplastic GPs were significantly higher than in patients with fundic glandular polyps and adenomatous polyps, suggesting that Hp infection was associated with the formation of inflammatory and hyperplastic polyps. These results were compatible with those of our study, where Hp infection was more prevalent in the inflammatory polyps group than in the fundic glandular polyps group.

There were some limitations to this study. First, the cross-sectional nature of the survey does not support the assumption of a causal relationship. Second, this study was not a randomized controlled study; because it was a retrospective study using clinical data, complete information was not available on certain baseline data such as weight, body mass index, and serum pepsinogen ratio of the participants. Third, the current study only included healthy people who were referred for a gastroscopy; people without medical insurance or younger people might be less likely to undergo gastroscopy. Fourth, not all cases had both a completed pathological examination and the Hp tests. Fifth, the study was conducted in a tertiary endoscopic center, so the possibility of a selection bias cannot be ignored.

## Conclusion

In summary, since the results of the present study showed that the incidence of abnormal upper gastrointestinal endoscopic results is high in healthy people undergoing a check-up, as well as indicated an association between abnormal upper gastrointestinal endoscopic results in healthy people, and sex, age and Hp infection, we suggest that gastroscopy be part of a routine medical examination for healthy subjects. Periodic physical examination is an effective way to monitor and manage physical health.

## Data Availability

The datasets used and/or analysed during the current study are available from the corresponding author on reasonable request.

## Abbreviations

Hp: *Helicobacter pylori*
UBT: urea breath test
GPs: gastric polyps
